# Linguistic validation of the Alberta Context Tool and two measures of research use, for German residential long term care

**DOI:** 10.1186/1756-0500-7-67

**Published:** 2014-01-31

**Authors:** Matthias Hoben, Marion Bär, Cornelia Mahler, Sarah Berger, Janet E Squires, Carole A Estabrooks, Andreas Kruse, Johann Behrens

**Affiliations:** 1 Network Aging Research (NAR), Ruprecht-Karls-University Heidelberg, Bergheimer Str. 20, 69115 Heidelberg, Germany; 2 Institute of Health and Nursing Sciences, Medical Faculty, Martin-Luther-University Halle-Wittenberg, Halle (Saale), Germany; 3 Institute of Gerontology (IfG), Ruprecht-Karls-University Heidelberg, Heidelberg, Germany; 4 Department of General Practice and Health Services Research, University Hospital, Ruprecht-Karls-University Heidelberg, Heidelberg, Germany; 5 School of Nursing, Faculty of Health Sciences, University of Ottawa, Ottawa, Canada; 6 Clinical Epidemiology Program, Ottawa Hospital Research Institute, Ottawa, Canada; 7 Faculty of Nursing, University of Alberta, Edmonton, Canada

**Keywords:** Cognitive debriefing, Translation, Alberta context tool, Estabrooks’ kind of research utilization items, Conceptual research utilization scale, Organizational context, Research utilization, Residential long term care

## Abstract

**Background:**

To study the association between organizational context and research utilization in German residential long term care (LTC), we translated three Canadian assessment instruments: the Alberta Context Tool (ACT), Estabrooks’ Kinds of Research Utilization (RU) items and the Conceptual Research Utilization Scale. Target groups for the tools were health care aides (HCAs), registered nurses (RNs), allied health professionals (AHPs), clinical specialists and care managers. Through a cognitive debriefing process, we assessed response processes validity–an initial stage of validity, necessary before more advanced validity assessment.

**Methods:**

We included 39 participants (16 HCAs, 5 RNs, 7 AHPs, 5 specialists and 6 managers) from five residential LTC facilities. We created lists of questionnaire items containing problematic items plus items randomly selected from the pool of remaining items. After participants completed the questionnaires, we conducted individual semi-structured cognitive interviews using verbal probing. We asked participants to reflect on their answers for list items in detail. Participants’ answers were compared to concept maps defining the instrument concepts in detail. If at least two participants gave answers not matching concept map definitions, items were revised and re-tested with new target group participants.

**Results:**

Cognitive debriefings started with HCAs. Based on the first round, we modified 4 of 58 ACT items, 1 ACT item stem and all 8 items of the RU tools. All items were understood by participants after another two rounds. We included revised HCA ACT items in the questionnaires for the other provider groups. In the RU tools for the other provider groups, we used different wording than the HCA version, as was done in the original English instruments. Only one cognitive debriefing round was needed with each of the other provider groups.

**Conclusion:**

Cognitive debriefing is essential to detect and respond to problematic instrument items, particularly when translating instruments for heterogeneous, less well educated provider groups such as HCAs. Cognitive debriefing is an important step in research tool development and a vital component of establishing response process validity evidence. Publishing cognitive debriefing results helps researchers to determine potentially critical elements of the translated tools and assists with interpreting scores.

## Background

Substantial evidence suggests that residential long term care (LTC) providers’ use of best practices is sub-optimal in Germany [1-7]. Research implementation is complex, challenging and hard to manage [8-10]. Organizational context (i.e., “the environment or setting in which the proposed change is to be implemented” [11] (p. 150), or–more generally–“the environment or setting in which people receive health care services” [12] (p. 96)) has been argued to be of vital importance in these processes [9,13-22]. However, research implementation and its influencing factors are not well understood in the residential LTC setting [23-26].

We lack instruments in German that a) capture reliable and valid scores on organizational context and research utilization in residential LTC institutions, and b) can be used with various provider groups in this setting. We thus translated three Canadian tools into German (see [27] for details): the Alberta Context Tool (ACT) [28-30], the Estabrooks’ Kinds of Research Utilization (RU) items [31,32] (residential LTC version [18]) and the Conceptual Research Utilization (CRU) Scale [33]. These tools have been widely used to investigate health care providers’ utilization of research in their daily work and its association with organizational context [18,33-35]. The psychometric properties of the three instruments have been evaluated, providing substantial evidence for appropriate acceptability, reliability and validity. See [36] for the ACT pediatric acute care version, based on RN responses; [20] for the ACT residential LTC version, based on health care aide (HCA) responses; [35] for an overview of studies assessing the psychometric properties of the Estabrooks’ Kinds of RU items; and [33] for the CRU Scale, based on HCA responses.

The ACT contains 10 concepts of organizational context: (1) leadership, (2) culture, (3) evaluation (feedback processes), (4) social capital, (5) informal interactions, (6) formal interactions, (7) structural and electronic resources, (8) organizational slack (staff), (9) organizational slack (space) and (10) organizational slack (time) [34]. Three versions (acute care–adult hospitals and pediatrics, residential LTC, community health) are available, containing forms for six provider groups (HCA, registered nurses (RN), allied health professionals (AHP), practice specialists, managers, physicians). The English original was translated into four languages (Dutch, Swedish, Mandarin Chinese, French) [34]. In our study we translated the HCA, RN, AHP, specialist, and manager forms of the ACT LTC version into German [27].

The Estabrooks’ Kinds of RU tool [31,32] comprises four items, each of them reflecting a particular kind of research utilization: (1) instrumental (i.e., using observable research-based practices when caring for residents), (2) conceptual (i.e., thinking about research-based knowledge and then using it to inform clinical decision making), (3) persuasive (i.e., using research findings to win an argument or make a case to someone), and (4) overall (i.e., using any kind of research findings, in any kind of way, in any aspect of work). Items ask care providers how often they used research in the described way. In the HCA questionnaires, the conceptual RU item is not included. The CRU Scale is a one-concept, five-item tool, asking care providers how often best practice knowledge e.g., gave them new knowledge or changed their mind [33].

The aim and challenge of the translation process is to ensure validity of scores obtained with the translated instruments. We need to maintain the quality of source instruments (ensuring equivalence of source and target versions) and, simultaneously, ensure that translated instruments are appropriate for the target audiences (meeting adaptation needs) [37,38]. In a previous publication, we reported on the translation process, the challenges and the strategies chosen to deal with challenges [27]. In this paper we, report on the cognitive debriefing–a linguistic validation procedure to “assess the clarity, intelligibility, appropriateness, and cultural relevance of the target language version to the target population” [39] (p. 47). This is a critical step in translating assessment instruments, as it examines how the target audience responds to translated items and whether they understand them as intended by the tool developers [40,41].

The evidence provided by cognitive debriefing corresponds to response process validity evidence, as defined by the standards for educational and psychological testing [42] (hereafter referred to as “the standards”). These standards are regarded as best practice in psychometric testing [43] and they guided our understanding of validity. In contrast to approaches which suggest that there are different types of validity (e.g., construct or criterion validity), the standards regard validity as a “unitary concept” [42] (p. 11) for which different sources of evidence are available: (1) tool content, (2) response processes, (3) internal structure and (4) relations to other variables. Validity then is “the degree to which all the accumulated evidence supports the intended interpretation of test scores for the proposed purpose” [42] (p. 11). Content evidence is obtained if tool items represent the construct(s) the tool intends to measure. We obtained initial content validity evidence through an expert panel step in the translation process [27]. Response process evidence indicates whether the test participants understand tool items as intended by the tool developers. Internal structure evidence refers to the associations between the tool items and components and their conformity with the proposed construct(s). Relations to other variables evidence is supported if the tool items are related (or not related) to concepts to which they are theorized to be related (or not related). In this article we present our cognitive debriefing results, providing information about response process validity evidence of the translated tools.

## Methods

**Figure 1:**
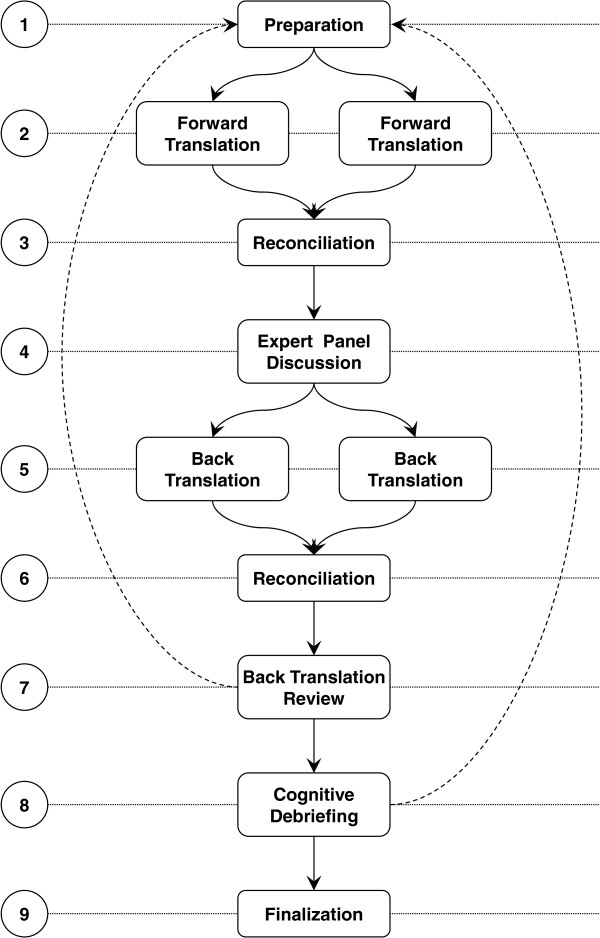
Steps of the translation process.

Cognitive debriefing was one of the last steps in the translation process (Figure 1, step 8, see [27] for details). Translation guidelines state that cognitive debriefing is an important step in instrument translation, but guidelines differ in the suggested methods and the level of detail of the instructions [38,44]. We identified a need for additional information to design this step, as the guidelines we used in our translation process [38,44] did not cover all methodological questions that arose. We based our design on Willis’ [41] comprehensive overview of cognitive interviewing methods. Table 1 shows the critical issues in designing the cognitive debriefing process, potential options and our decisions.

**Table 1:** Critical issues, potential options and decisions made in designing the cognitive debriefings

**Sample**	
Participants	• One facility versus **multiple facilities**
	• **Sampling criteria for individuals**
Items	• All questionnaire items versus **selected items**
	• **Item selection criteria**
**Data collection**	
Technique	• Think-aloud
	• **Verbal probing**
	• Vignettes, card sorts, field-based probes
Details	• Concurrent probing versus **retrospective probing**
	• Standardized probes versus free-form probes **(combination used in this study)**
	• Proactive probing versus reactive probing **(combination used in this study)**
	• **Individual interviews** versus focus groups
	• **Face-to-face interviews** versus telephone interviews
Documentation	• Tape recording versus field protocol **(combination used in this study)**
**Analysis**	
Details	• Interview data versus field protocols **(combination used in this study)**
	• Literal transcription versus **condensed statements**
	• Deductive analysis using a predefined coding scheme versus inductive development of the codes **(combination used in this study)**
	• Frequency of problems per question versus type and quality of problem(s) **(combination used in this study)**

Note: Boldface indicates the options chosen in this study.

### Ethics approval

The study was approved by the Ethics Committee of the Medical Faculty, Martin-Luther-University Halle-Wittenberg, Germany (reference number: 2011–39).

### Sample

Cognitive debriefing is a method to pretest and validate a survey questionnaire, involving qualitative interview methods and thus typically including small samples of 5 to 15 target group participants [39,41]. Our care provider sample consisted of 39 participants from a convenience sample of five residential LTC facilities: 16 HCAs, 5 RNs, 7 AHPs, 5 specialists and 6 managers. Ten HCAs participated in the initial cognitive debriefing. We modified the translated instruments based on the findings from this round and tested them again in a second cognitive debriefing round with three other HCAs. As this step led to further item modifications, we carried out a third cognitive debriefing round with another three HCAs. Only one cognitive debriefing round was needed for the other provider groups. The inclusion and exclusion criteria for care providers are shown in Table 2.

**Table 2:** Inclusion and exclusion criteria for care providers in the cognitive debriefing

**Inclusion criteria**	**Exclusion criteria**
Employed in a residential LTC institution recognized by the German social law	Volunteers, temporary workers
Working in the LTC facility for >3 months	Working in the LTC facility for <3 months
At least 25% of a full-time job	Less than 25% of a full time job
Sufficient German language skills to understand German ACT and RU wording	German language skills are insufficient to understand ACT and RU wording

Cognitive debriefing focusses on studying “the cognitive processes that respondents use to answer survey questions; in particular their *comprehension, recall, decisions and judgement,* and *response processes*” [41] (p. 6; emphases in the original). In order “to detect a wide range of problems in survey questionnaires” [41] (p. 6), samples should include participants with a broad spectrum of characteristics, known or expected to influence item understanding [39,41]. Variation, achieved by judgement sampling (i.e., actively selecting the most productive sample with regard to those essential characteristics) [45], is therefore more critical for cognitive debriefing samples than statistical representativeness [39,41]. Characteristics known to be important include: age, sex, level of education, and socio-economic background [39,41]. In addition, Squires et al. [33] found that HCAs whose native language was not English responded differently to the Items of the CRU Scale than English native speakers – underscoring the significance of ethnicity and native language for item understanding. The managers of the participating facilities were asked to identify eligible staff members and ask them if they would like to participate. In order to reflect the heterogeneity of care providers in the residential LTC setting, we sampled the participants according to characteristics potentially influencing their abilities to understand tool items (Table 3).

**Table 3:** Sampling criteria

**General**	
Language skills	• Native language not German and moderate German language skills
	• Native language not German and good German language skills
	• Native language German
Job experience	• Little: <1 year
	• Moderate: 1–4 years
	• High: 5–9 Years
	• Very high >10 years
General education^1^	• *Haupt-/Volksschule* (lowest school level, ends after the 9th grade)
	• *Realschule* (Intermediate school level, ends after the 10th grade)
	• Vocational training
	• *Gymnasium* (highest school level, ends after the 12th or 13th grade)
	• Academic degree
**Provider group specific**	
Health care aides^1^	• No HCA training
	• HCA training
Registered nurses^2^	• Geriatric nurse
	• Adult acute care nurse
Allied health providers	• Therapist with academic training
	• Therapist with vocational training
	• Assistant with no vocational training
Specialists	• Quality manager
	• Clinical specialist
Managers	• Facility instructor
	• Nursing director
	• Unit leader

^1^In Germany HCAs can take one year of training and obtain an HCA degree, either for the care of elderly persons, for adult acute care or for pediatric care; only a few pediatric HCAs work in nursing homes, with none in this sample.

^2^In Germany there are three setting-specific registered nursing degrees: geriatric nurse for the care of elderly persons, adult acute care nurse and pediatric nurse; only a few pediatric nurses work in nursing homes, with none in this sample.

We began with the HCAs, asking their managers to identify HCAs who were eligible and willing to participate. First, we identified one HCA with a combination of criteria that we assumed would reflect a low ability to understand tool items: (1) native language not German but moderate German language skills, (2) little job experience (between three and six months), (3) low general education level, and (4) no HCA training. Next, we identified one person with a combination of criteria that we expected would maximize their ability to understand tool items: (1) native language German, (2) extensive job experience (>10 years), (3) high general education level, and (4) HCA training. Finally, we included eight persons with combinations of criteria somewhere between those of the first two HCAs. In rounds two and three of the HCA cognitive debriefing, we used the same procedure to identify new participants: identify one person with a criteria combination unfavourable for item understanding, one with a criteria combination optimal for item understanding, and one in between. RNs, AHPs, specialists and managers were sampled similarly.

### Item selection

Due to staff time constraints in residential LTC it was impossible to test all items with all participants. We thus selected a list of items for each participant before their interview and data collection. Participants completed the whole questionnaire (including all ACT and RU items), but only selected items were discussed in their interviews. Six ACT items (three items from the feedback sub-scale, two slack (time) items and one slack (space) item) and all Estabrooks’ Kinds of RU items had been difficult to translate and had been extensively discussed in the expert focus groups and back translation reviews. We thus included them in all item lists. The remaining HCA ACT and CRU items were randomly allocated to the lists of the 10 HCAs participating in the initial cognitive debriefing round, until a) each item was assigned to at least one list and b) each of those lists contained 20 items. Our approach of randomized item selection is an adaptation of the proceeding described by Schuman [46] as part of the random probe technique. In second and third HCA cognitive debriefing rounds we included only the items revised in the previous round. In the cognitive debriefing sessions with the other provider groups the items were not selected randomly. In all lists for participants from other provider groups we included the six ACT and Estabrooks’ Kinds of RU items as above, as well as ACT items that were problematic in the HCA cognitive debriefings and all ACT items that differed between the previously translated version and the one to be tested. Finally, all items from the CRU Scale were added. This resulted in lists containing 26 items for RNs, 25 items for allied health professionals, 24 items for specialists and 28 items for managers.

### Data collection

First, the researcher explained the procedure to the participant and asked for informed consent. Participants who were willing to participate completed the questionnaire. Subsequently, the researcher reviewed questionnaire responses for missing items or mistakes (e.g., items ticked twice or items ticked although they should have been skipped according to skip patterns). Participants were asked whether they found some items hard to understand or to answer, and how they rated the clarity of the questionnaire design. Problematic items were added to the predefined item lists if not already included.

After participants completed the questionnaire, we conducted individual cognitive debriefings. In the interviews, participant understanding of the items was assessed using verbal probing–a qualitative, semi-structured interview method. The interviewer stimulated participant reflections on the meanings of questionnaire items or the backgrounds of their answers to questionnaire items by asking specific types of questions–so-called cognitive probes [41]. Willis [41] discusses six kinds of probes, which we adapted. Each type of probe is illustrated in Table 4 with an example question based on one of the ACT items. That item asked participants to what extent they agree or disagree that they are a member of a supportive team. They could select one of five answers on a Likert scale: strongly disagree, disagree, neither agree nor disagree, agree or strongly agree.

**Table 4:** Six kinds of cognitive probes with example questions

**Cognitive probe**	**Example**
Comprehension/interpretation probe	What does “supportive team” mean to you?
Paraphrasing	Can you repeat the question I just asked in your own words?
Confidence judgment	How sure are you that you are (or are not) a member of a supportive team?
Recall probe	How do you remember your experiences with your team in the past week?
Specific probe	Why do you think you are (or are not) a member of a supportive team? Can you explain the background of your answer please?
General probes	How did you arrive at that answer? Was that easy or hard to answer? What did you think when answering this question?

We followed Willis’ [41] (p. 95) recommendation to maintain “a flexible approach to probe construction”. Before the interviews, we developed example questions for all six probes relating to each of the included tool items. We did not decide definitely which kind of probe to use with which item. As Willis [41] (p. 95) states, “the most interesting and productive forms of probing often develop through the course of the interview, as the product of the particular relationship between the interviewer, subject, and survey questionnaire”. Therefore, the interviewer used an interview guideline with example questions, but was free to choose the kind of probe and to ask questions other than the ones pre-formulated, depending on the tool item and communication situation. Participants responded to the probes in their own words with open ended statements. The interviews were recorded with an electronic voice recorder.

### Data analysis

Recorded interviews were transcribed and interview texts were reduced by MH using a qualitative content analysis technique called “summarizing content analysis” [47,48]. Text segments constituting a unit of meaning (typically sentences) and referring to participants’ responses to the cognitive probes were identified. Components not related to the core content (such as repetitions or embellishing elements) were removed and the remaining sentence was reduced to a concise statement by paraphrasing it. These statements were compared to concept maps, designed by the instrument developers, which define each concept in detail. Responses to each of the probed items were evaluated by MH and MB as to whether they matched the relating concept map definition.

### Revision of items and further cognitive debriefing rounds

**Figure 2:**
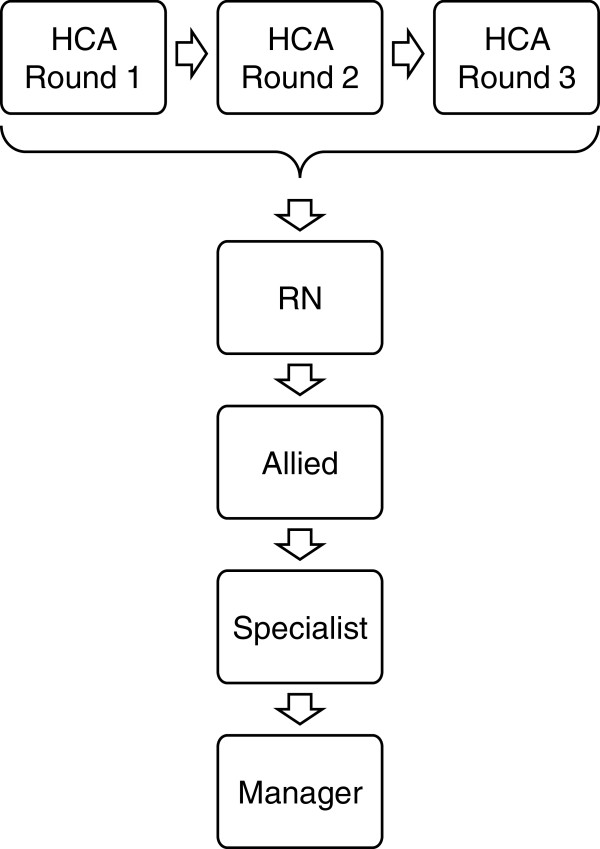
Sequence of the cognitive debriefing rounds.

Items were revised if answers from at least two participants did not match the relating concept map definition. The revised wording was then tested in another cognitive debriefing round. As Figure 2 shows, three rounds were required for the HCA forms before the participants understood all items as intended. In the other translations (RN, AHP, specialist, manager), participants understood all items in the initial cognitive debriefing.

## Results

### Sample description

Thirty-nine providers from five non-profit nursing homes in the “*Metropolregion Rhein-Neckar*” (in the south-west of Germany) participated. The median number of beds per facility was 163 (range = 82 to 217). The mean age of the participants was 40.26 years (SD = 10.58). Of the nine non-native German speakers, six specified Polish as their native language, two Russian, and one Spanish. Their mean number of years speaking German was 13.56 (range = 2.00 to 39.00, SD = 12.76). Further details of participants’ socio-demographic characteristics are given in Table 5.

**Table 5:** Socio-demographic characteristics of the cognitive debriefing sample (n = 39 providers)

**Characteristics**	**N**
**Sex**	
Female	35
Male	4
**Age**	
20–29 years	7
30–39 years	13
40–49 years	9
50–59 years	10
**Native language**	
German	30
Not German	9
**Job experience**	
<1 year	8
1–4 years	7
5–9 years	8
10–14 years	3
15–20 years	5
20–24 years	3
>24 years	5
**General education**	
*Haupt-/Volksschule* (lowest school level, ends after the 9th grade)	4
*Realschule* (Intermediate school level, ends after the 10th grade)	1
Vocational training	23
*Gymnasium* (highest school level, ends after the 12th or 13th grade)	7
Academic degree	4

### HCA cognitive debriefing

An item was classified as problematic if the answers from at least two participants did not match the intended item meaning. Table 6 provides an example of an answer that matched well with the intended item meaning, Table 7 contains an example of a problematic item.

**Table 6:** Example of a HCA answer matching with the intended item meaning

Wording of the original English item	We have *private space* such as a conference room on this unit or floor (*other than* at the bedside, in the hallway or medication room) to discuss resident care plans and share knowledge about resident care and best practices.
German wording approved for the cognitive debriefing	Wir verfügen über einen *nicht öffentlichen Personal- oder Besprechungsraum* auf diesem Wohnbereich oder Stockwerk. Diesen können wir nutzen, um über die Pflegeplanungen zu sprechen sowie um Wissen über die optimale Pflege und Betreuung der Bewohner auszutauschen.
English back translation of the German wording	We have use of a *private staff room or meeting room* in the residential care unit or on the floor. We can use this room to discuss resident care plans and to share knowledge about best-practices in caring for residents.
Researcher question (general probe)	“Which room did you have in mind? Can you describe it please?”
Participant answer	“Well, we have got two ones. One is our office here on the unit. And the second one is our so-called ‘break room’. If we really want to talk about care without interruption and don‘t want residents or family members to enter, we go there. If somebody wants anything, he needs to knock at the door before.”

**Table 7:** Example of a HCA answer not matching with the intended item meaning

Wording of the original English item	My organization effectively *balances* best practice and productivity.
German wording approved for the cognitive debriefing	Meine Einrichtung schafft erfolgreich *den Ausgleich* zwischen optimaler Pflege- und Betreuungsqualität und Wirtschaftlichkeit.
English back translation of the German wording	My organization successfully *manages the balance* between optimal care quality and cost effectiveness.
Researcher question (paraphrasing)	“Can you repeat the question in your own words?”.
Participant answer	“If we are able to be there for the residents and to give them the best possible care—the care they want and need”.
Researcher question (comprehension/interpretation probe)	“What does ‘*Ausgleich*’ [‘balance’] mean to you?”.
Participant answer	“To take into account resident‘s habits and to do things as he used to do them”.
Researcher question (comprehension/interpretation probe)	“OK, and how did you understand ‘*Wirtschaftlichkeit*’ [‘productivity’]?”.
Participant answer	“Well, I can‘t do anything with it. No idea”.

In the first example, the participant understood that the question related to a private room, used to talk about resident care or best practices. The second example shows that the participant only focussed on the best practices part of the question. She understood neither the concept of balancing nor the concept of productivity as the counterpart to be balanced with best practice.

Results of the first round of HCA cognitive debriefing demonstrated that 11 of the 58 ACT items and all 8 RU items were not understood by at least two of the participants. Examples of these items and their revisions are presented in Additional file 1.

Based on these results, 4 of the 11 problematic ACT items and all RU items were modified. We decided not to modify the wording of the formal interactions item (continuing education (…) *outside* this nursing home). Two participants did not read carefully enough and thought about education *in* their facility. Wording modification would not have resolved that issue. We also did not modify the six feedback items, which ask the participants how often they get formal information about care quality and how this information is handled in their facility. The problem was not caused by the items themselves, but rather by the preceding stem, which we therefore changed. Participants did not think about overarching information for their entire unit or facility (such as falls rate), but rather about information related to individual residents. Therefore the answers to all feedback items were incorrect. Changes to the stem aimed for more clarity about which kind of information was required for the items. In the English version, the RU items ask the HCAs how often they used best practices in specific ways in their daily routine. In German there is no corresponding word for best practice. Germans often use the English term, but the HCAs were not familiar with this. As a result, we chose to describe this principle and to provide examples for the sake of clarity (see [27] for further details). The changed wordings are presented in Additional file 1.

After analysis of the round two data, 2 of the 4 modified ACT items were still problematic: culture item 3 and structural and electronic resources item 5 (Additional file 1). In addition, the six ACT feedback items and all 8 RU items were still problematic. In order to have the participants focus on the intended kind of information, we changed the feedback stem again by (1) introducing the term “statistics”, (2) emphasizing the important passages even more, and (3) adding a sentence that explained what was not meant by this item (i.e., individual resident information). The other two problematic ACT items and the RU items were also further modified. We described the best practice term by the wording “knowledge of how to provide the best possible care quality” (German: “*Fachwissen über optimale Pflege und Betreuung*”). In round three, all items were understood as intended.

### Cognitive debriefing with the other provider groups

In the German questionnaires for the other provider groups, we adopted the new item wordings that we had developed during the HCA cognitive debriefing sessions for all items, which had the same wording in the original Canadian tools for both the HCA version and the versions for the other providers: the two Culture items, Feedback stem and all six items, and the Structural and Electronic Resources item (Additional file 1). The English wording of the other items (ACT Time item and the RU tools items) differs in the Canadian tools between the HCA questionnaires and the questionnaires for the other provider groups. The term “best practice” is used for the HCAs, while “clinical knowledge” (ACT Time item) or “research use“ (RU tools) was chosen for the other provider groups. We retained this difference in our translation and used the German wording “*klinische Erkenntnisse*” (clinical knowledge, ACT time item) and “*Anwendung wissenschaftlicher Erkenntnisse*” (use of scientific knowledge, RU tools) for the regulated providers. In the subsequent cognitive debriefings with RNs, AHPs, specialists and managers, we included all items of the two RU tools, all items that were problematic in the translation process, all items that were problematic in the HCA cognitive debriefing and all items whose wordings differed between the previously translated version and the one to be tested (see Methods section for details). All participants understood each of the included items as intended. Thus, only one cognitive debriefing round was required for each of these provider groups. An example answer of each provider group to the instrumental research use item is provided in Table 8.

**Table 8:** Examples of answers of RNs, AHPs, specialists and managers to the instrumental research use item

Wording of the original English item	**Definition:** Using observable research-based practices when caring for residents. By this we mean *practice* that may be guided by guidelines, protocols, routines, care plans or procedures that are based on research.
	**Question:** On your **LAST typical work day**, how often did you use research in this way?.
German wording approved for the cognitive debriefing	**Definition:** Beobachtbare pflegerische Handlungen durchführen, die auf wissenschaftlichen Erkenntnissen basieren. Damit meinen wir *Handlungen*, für die Sie forschungsbasierte Vorgaben wie Standards, Leitlinien, Richtlinien oder Verfahrensanweisungen haben.
	**Question:** An Ihrem **LETZTEN typischen Arbeitstag**: Wie häufig haben Sie wissenschaftliche Erkenntnisse auf diese Weise angewendet?.
English back translation of the German wording	**Definition:** To perform observable care interventions based on evidence based findings. By this we mean interventions for which there are research based guidelines such as standards, policies and protocols, or procedure guidelines.
	**Question:** On your **LAST typical day of work**: how often did you use research in this way?.
**RN example**	
Participant answer to questionnaire item	Almost 100% of the time.
Researcher question (general probe)	“Can you please give reasons for your answer?”.
Participant answer	“Well, I am able to do that very often. But of course the price is high. It is a matter of our personal health. I always try to comply with the guidelines 100%. That is never just 50%. Wound management, pressure ulcer prevention, challenging behaviour – all the topics you have listed here”.
Researcher question (confidence probe)	“How sure are you that your practice complies with the current state of knowledge?”.
	“Well, we have an expert for each of those topics. Wound management for example, we had a training course just yesterday. Our knowledge is always on the most current level. I am completely sure about this”.
**AHP example**	
Researcher question (paraphrasing)	“Can you please state in your own words what the definition means to you?”.
Participant answer	“That care is being carried out properly, that no dangerous care is being provided. That is how I understand this. And that it follows the guidelines and instructions of how to care for a human being, that the person is treated and cared for accordingly.”
**Specialist example**	
Researcher question (paraphrasing)	“Can you please try to explain in your own words how you understood the definition?”.
Participant answer	“Well, I find it is explained very good. I have got guidelines, standards, instructions anything that is written in our quality handbook. That is scientific … scientific knowledge broken down for practice and implemented into our routines. And that is exactly what I was thinking about. There is, for example, a standard for pressure ulcer prevention – and how we have implemented that one into our practice”.
**Manager example**	
Participant answer to questionnaire item	10% or less of the time.
Researcher question (confidence probe)	“How sure are you that your answer is correct?”.
Participant answer	“I am doing all those things – pressure ulcer prevention, dealing with challenging behaviour etc. But that I use scientific knowledge for this does not happen very frequently”.

## Discussion

The role of organizational context must be understood to improve research implementation in residential LTC, but that understanding is still lacking [23-25]. Researchers need robust assessment tools to study organizational context [49,50]. We could find no German assessment tool that a) specifically and validly assessed modifiable organizational context factors that are asserted to influence research implementation in residential LTC, and b) could be used with various residential LTC provider groups. Thus, we translated three Canadian tools [27] from English into German. Cognitive debriefing is an essential step to assess response process validity of the translated tools [40-42].

Finding an appropriate German wording for items asking HCAs about best practices (all 8 RU items, ACT culture item 3, and ACT time item 3) was the major challenge for us, particularly in the RU tools translations. While “research” is the wording of choice in the regulated provider RU tool versions (RNs, AHPs, specialists, managers), “best practice” is used in the HCA forms, as this terminology is commonly used and better understood by English-speaking HCAs [30,33,51,52]. German has no equivalent for this term and German HCAs didn’t understand the English term when it was directly adopted in German. Furthermore, German HCAs had difficulty understanding the terms “research”, “research knowledge”, “scientific knowledge” or “evidence”. They often found it hard to imagine what kind of research knowledge might be available and relevant for their practice. Like Canadian and Swedish residential LTC providers [51], they tended to discuss barriers to RU rather than RU itself. However, they agreed that some kind of knowledge is important for their practice–either obtained by experience or by asking colleagues. This is consistent with findings that even RNs prefer informal, interactive or experience-based knowledge sources to formal ones such as journals or text books [53-55].

Nevertheless, we were highly motivated to find wording that HCAs understood. In Germany, about 40% of the staff providing direct care in residential LTC are HCAs (i.e., staff with one year of HCA training, brief training of a few weeks or months, or no elder-care-related training at all) [56]. They give feeding assistance, mobilize residents, turn them to prevent pressure ulcers, provide oral health care, interact with persons with dementia, etc. All of these tasks can pose safety risks to residents if carried out improperly. We thus believe that it is crucial to know how HCAs rate their use of best practice. Very few RU studies have included HCAs as yet [33,57-59]. The rigorous translation process [27] and the cognitive debriefing in particular were important to create robust tools. The cognitive debriefing helped us to detect problematic items that would have undermined the validity of the tools’ scores if unmodified. In our context, it was essential to avoid specific instrument terms like “research”, “scientific”, or “best practice” in favour of clear simple terms and explanations of these concepts. The translation of “best practice” to “knowledge of how to provide the best possible care quality” (German: “… Fachwissen über optimale Pflege und Betreuung …”) worked best and facilitated HCAs’ comprehension and understanding of those items.

We expected that the problems discussed above would occur mainly with the HCAs, and that the regulated providers would understand the more technical wording. Therefore, we did not adopt the HCA wording “best practice” for items referring to “research use” in the regulated provider versions. Revised versions of the remaining problematic HCA items, which we assumed would also be problematic in the other provider groups (i.e., the individual versus unit/facility level problem of the ACT Feedback section), were adopted in the regulated provider forms. Working on the items and modifying them until the HCAs understood them was certainly time consuming but necessary and ultimately fruitful. All items understood by the HCAs were subsequently understood by the other provider groups, indicating no further need for modifications.

The cognitive debriefing step helped us prepare the instruments for our larger field testing study. We are evaluating the psychometric properties of the translated instruments and the relationship between organizational context and research implementation in German residential LTC facilities. Qualitative findings will assist us as well as future researchers in interpreting the instrument scores and identifying and understanding potential problems. However, additional validity evidence sources (i.e., internal structure and relations to other variables) need to be examined. Currently, we are evaluating the translated tools with this focus in a larger sample.

Some limitations of cognitive interviewing need to be considered. Generally, cognitive interviewing tends to underestimate problems because:

(1) persons who volunteer to participate in cognitive interviews are more ready to spend time thinking about the items, are often better educated and are more confident in being able to understand the questionnaire.

(2) it is a testing situation in which participants work to perform well and are “patient and forgiving” [41] (p. 226).

In addition, we did not test all items with each participant, a condition that we accepted for both better feasibility of the cognitive debriefing and participants’ compliance. Thus, we tested some problematic items with all participants and distributed all other items randomly among participants, ensuring that no item remained untested and avoiding selection bias. Finally, due to the qualitative design the sample size was relatively small (although appropriate for cognitive debriefing purposes). Facilities and care providers participating in our study are therefore not statistically representative of the German facility and care provider population. Although we could find evidence for response process validity of the final questionnaires, these results cannot be generalized. Validity needs further investigation in larger samples, using rigorous statistical methods. Nevertheless, our cognitive debriefings detected a variety of problems and helped to minimize them, although other problems may remain.

## Conclusions

Cognitive debriefing is essential in translating instruments as an early step in instrument validation. It provides information about response process validity evidence and helps translators to detect and respond to problems. Translating tools intended to assess HCA use of research is challenging. HCAs are not trained to find and use research on their own and they are not familiar with the related terminology. However, assessing their use of best practice is important because they provide hands-on care that may risk the safety of residents if not provided properly. Cognitive debriefing is important to assess whether HCAs understand the chosen wording of tool items, in order to validly assess their rating of best practice use. Publishing cognitive debriefing results helps researchers anticipate and plan for potential challenges, determine potentially critical elements of the translated tools and interpret the resulting scores.

## Abbreviations

ACT: Alberta context tool; AHP: Allied health professional; CRU: Conceptual research use; HCA: Health care aide; LTC: Long term care; RN: Registered nurse; RU: Research use.

## Competing interests

The authors declare that they have no competing interests.

## Authors’ contributions

MH led the development of the translation process and cognitive debriefing design, led the research project, carried out the cognitive debriefings, and drafted this manuscript. MB contributed to the design and analysis of the cognitive debriefings, and was a member of the translation team (second forward translator). CM assisted with the development of the translation process and cognitive debriefing design and performed the first back translation. SB was member of the translation team (second back translation). CAE and JES developed the ACT and the two RU measures, approved the translation of the tools, did the back translation reviews and provided advice and recommendations regarding the translation process and cognitive debriefing design and the translations. AK and JB were the co-supervisor and primary supervisor, respectively, of the entire project. All authors contributed to the research design and contributed to, read, and approved the final version of this manuscript.

## Supplementary Material

Additional file 1 Problematic items in the HCA cognitive debriefings and revision history. Click here for file
