# Supplementary material for: Linguistic validation of the Alberta Context Tool and two measures of research use, for German residential long term care
Source: BMC Res Notes. 2014 Jan 31;7:67. doi: 10.1186/1756-0500-7-67 (PMC3913324; doi:10.1186/1756-0500-7-67)
Supplement: Additional file 1 — Problematic items in the HCA cognitive debriefings and revision history. [file 1756-0500-7-67-S1.pdf]

Additional File 1: Problematic items in the HCA cognitive debriefings and revision history

ACT					
**Concept**	**Item #**	**Original wording**	**Wording round 1**	**Wording round 2**	**Wording round 3**
**Culture**	3 and 6	**Example item:**<br>My organization effectively *balances* best practice and productivity.	**German:**<br>Meine Einrichtung schafft erfolgreich *den Ausgleich* zwischen optimaler Pflege- und Betreuungsqualität und Wirtschaftlichkeit.<br>**English:**<br>My organization successfully *manages the balance* between optimal care quality and cost effectiveness.	**German:**<br>Meiner Einrichtung gelingt es, optimale Pflege und Betreuungsqualität und finanzielle Interessen *in Einklang* zu bringen<br>**English:**<br>My organization is successfully *manages to bring* optimal care quality *in accordance* with financial interests	**German:**<br>Meiner Einrichtung gelingt es, *sowohl* optimale Pflege- und Betreuungsqualität sicherzustellen *als auch* wirtschaftlich zu arbeiten (d.h. die zur Verfügung stehenden Mittel - z.B. Zeit, Geld, Personal etc.) optimal einzusetzen).<br>**English:**<br>My institution successfully *manages the balance* between optimal care quality and working cost effectively (which means to use the available resources – e.g. time, money, staff, etc. – optimally)
**Feedback**	Stem	Sometimes you may receive formal information about the care that is being provided to residents. Examples of this kind of information include: *number of resident falls and pain control.*	**German:**<br>Vielleicht erhalten Sie gelegentlich offiziell (schriftlich oder mündlich) Auskunft über die Pflege- und Betreuungsqualität auf Ihrem Wohnbereich. Beispiele für solche Informationen sind: *Zahl der Bewohner, die gestürzt sind oder einen Dekubitus haben*.<br>**English:**<br>You may sometimes receive official (written or oral) information about the care quality in your residential care unit. Examples of such information are: *the number of residents that have had falls or developed pressure areas*.	**German:**<br>Vielleicht erhalten Sie gelegentlich offiziell (schriftlich oder mündlich) Rückmeldung über die Pflege- und Betreuungsqualität auf Ihrem Wohnbereich. Gemeint sind Statistiken für den gesamten Wohnbereich und für einen bestimmten Zeitraum (z.B. die letzten drei oder sechs Monate). Beispiele für solche Statistiken sind: *Zahl der Bewohner, die gestürzt sind oder einen Dekubitus haben*.<br>**English:**<br>You may sometimes receive official (written or oral) feedback about the care quality in your residential care unit. By this we mean statistics for your whole unit and for a specific period (e. g. the last three or six months). Examples of such **statistics** are: *the number of residents that have had falls or developed pressure areas*.	**German:**<br>Vielleicht erhalten Sie gelegentlich offiziell (schriftlich oder mündlich) Rückmeldung über die Pflege- und Betreuungsqualität auf Ihrem Wohnbereich. ***Gemeint sind <u>ausschließlich</u> Statistiken für den gesamten Wohnbereich und für einen bestimmten Zeitraum (z.B. die letzten drei oder sechs Monate). Nicht gemeint sind Informationen über einzelne Bewohner (z.B. in der Übergabe)***. Beispiele für solche ***Statistiken*** sind: *Zahl der Bewohner, die gestürzt sind oder einen Dekubitus haben*.<br>**English:**<br>You may sometimes receive official (written or oral) information about the care quality in your residential care unit. ***By this we mean <u>exclusively</u> statistics for your whole unit and for a specific period (e.g. the last three or six months). We do not mean information about individual residents here (e. g. during handover)***. Examples of such ***statistics*** are: *the number of residents that have had falls or developed pressure areas*.

ACT					
**Concept**	**Item #**	**Original wording**	**Wording round 1**	**Wording round 2**	**Wording round 3**
	1–6	**Example item:**<br>I routinely receive information on my team's performance on data *like* the examples provided above.	**German:**<br>Ich erhalte regelmäßig Informationen über die Arbeitsleistung meines Teams in Form von Daten, *ähnlich* der oben genannten Beispiele.<br>**English:**<br>I receive regular information about the performance of my team in the form of data *similar* to the above mentioned examples.	[Wording not changed]	**German:**<br>Ich erhalte regelmäßig Informationen über die Arbeitsleistung meines Teams in Form von **Statistiken**, *ähnlich* der oben genannten Beispiele.<br>**English:**<br>I receive regular information about the performance of my team in the form of ***statistics*** *similar* to the above mentioned examples.
**Formal Interactions**	4	Continuing education (conferences, courses) *outside* this nursing home?	**German:**<br>Fort- und Weiterbildung (z.B. Tagungen/Kongresse oder Schulungen) *außerhalb* Ihrer Pflegeeinrichtung?<br>**English:**<br>Continuing education (e.g. conferences, seminars or training courses) *external* to your care organization?	[Wording not changed]	[Wording not changed]
**Structural and Electronic Resources**	5	Policies and procedures for your facility (print/on-line)?	**German:**<br>Prozessvorgaben und Verfahrensanweisungen Ihrer Einrichtung (in gedruckter oder elektronischer Form)?<br>**English:**<br>Policies and procedural instruction documents from your organization (in printed or electronic form)	**German:**<br>Verfahrensanweisungen und Vorgaben Ihrer Einrichtung (in gedruckter oder elektronischer Form)?<br>**English:**<br>Policies and procedural instruction documents from your organization (in printed or electronic form)	**German:**<br>Verfahrensanweisungen und Vorgaben Ihrer Einrichtung (z.B. Hygienegrundsätze oder Dokumentationsvorgaben in gedruckter oder elektronischer Form)?<br>**English:**<br>Policies and procedural instruction documents from your organization (e. g. infection control principles or documentation instructions in printed or electronic form)?
**Time**	3	[How often do you] have time to talk to someone about best practices*?*	**German:**<br>[Wie oft haben Sie] Zeit, sich mit jemandem über den neuesten Erkenntnisstand zur optimalen Pflege und Betreuung zu unterhalten?<br>**English:**<br>[How often do you have] time to talk with someone about best-practices in caring for residents?	**German:**<br>[Wie oft haben Sie] Zeit, sich mit jemandem über neues Fachwissen zur optimale Pflege und Betreuung zu unterhalten?<br>**English:**<br>[How often do you have] time to talk with someone about best-practices in caring for residents?	[Wording not changed]

Estabrooks' Kind of RU tool					
**Concept**	**Item #**	**Original wording**	**Wording round 1**	**Wording round 2**	**Wording round 3**
**Instrumen-tal, Persua-sive and Overall RU**	1–3	**Example item:**<br>On your **LAST typical work day**, how often did you use this type of best practice knowledge to provide resident care?	**German:**<br>Wenn Sie an Ihren **LETZTEN typischen Arbeitstag** denken: Wie häufig haben Sie diese Art Wissen zur optimalen Gestaltung der Pflege und Betreuung von Bewohnern angewendet?<br>**English:**<br>On your **LAST typical day of work**: how often did you use best practice knowledge corresponding to the above mentioned examples, in any type or form at your work?	**German:**<br>Wenn Sie an Ihren **LETZTEN typischen Arbeitstag** denken: Wie häufig haben Sie wissenschaftliche Erkenntnisse der oben genannten Art (so genanntes „best-practice-Wissen“) bei der Pflege und Betreuung von Bewohnern angewendet?<br>**English:**<br>On your **LAST typical day of work**: how often did you use scientific knowledge (so called best practice knowledge) corresponding to the above mentioned examples, in any type or form at your work?	**German:**<br>Erinnern Sie sich jetzt bitte an die eben gelesenen ***Beispiele.***<br>Wenn Sie an Ihren **LETZTEN typischen Arbeitstag** denken: Wie häufig haben Sie solches oder ähnliches Fachwissen (siehe ***Beispiele***) bei der Pflege und Betreuung von Bewohnern angewendet?<br>**English:**<br>Please remember the above mentioned ***examples*** now.<br>On your **LAST typical day of work**: how often did you use best practice knowledge corresponding to the above mentioned ***examples***, in any type or form at your work?

CRU scale					
**Concept**	**Item #**	**Original wording**	**Wording round 1**	**Wording round 2**	**Wording round 3**
**Conceptual RU**	1–5	**Example item:**<br>[How often did best practice knowledge] give you new knowledge about how to care for residents.	**German:**<br>Sie haben neues Wissen darüber erlangt, wie Sie die Pflege und Betreuung der Bewohner gestalten können.<br>**English:**<br>You have obtained new knowledge as to how to provide care for residents.	**German:**<br>Sie wissen jetzt Neues darüber, wie Sie Bewohner pflegen und betreuen können.<br>**English:**<br>You now know new things about how to provide care for residents.	[Wording not changed]
